# Birth and parental characteristics and risk of neuroblastoma in a population-based Norwegian cohort study

**DOI:** 10.1038/sj.bjc.6604646

**Published:** 2008-09-02

**Authors:** T Bjørge, A Engeland, S Tretli, I Heuch

**Affiliations:** 1 Department of Public Health and Primary Health Care, University of Bergen, N-5020 Bergen, Norway; 2 Division of Epidemiology, Norwegian Institute of Public Health, N-5020 Bergen/N-0403 Oslo, Norway; 3 Cancer Registry of Norway, Institute of Population-based Cancer Research, Montebello, N-0310 Oslo, Norway; 4 Department of Mathematics, University of Bergen, N-5008 Bergen, Norway

**Keywords:** perinatal factors, neuroblastoma, cohort study, Norway

## Abstract

In this population-based Norwegian cohort study (2.1 million children), the impact of birth and parental characteristics on the risk of neuroblastoma (178 cases) was evaluated. In children below the age of 18 months, there was an increased neuroblastoma risk among those with congenital malformations and suggestion of increased risk when the mother had pre-eclampsia.

Neuroblastoma is the most common cancer in children less than 1 year of age, and is the third most common malignancy in childhood (below 5 years of age) in the United States (Goodman *et al*, 1999). The incidence of neuroblastoma has increased in some European countries during 1978–1997 (Kaatsch *et al*, 2006; Spix *et al*, 2006), possibly influenced by changes in identification and reporting of the disease over time. Although there has been substantial improvement in prognosis of well-defined subsets of patients in the past few decades, the long-term survival for children with high-risk disease is still less than 40% (Maris *et al*, 2007).

Neuroblastoma is an embryonal tumour that originates from primordial neural crest cells that eventually develop into the sympathetic nervous system (SNS) and the adrenal medulla. The tumour almost exclusively occurs in infants and young children. About 50% of the tumours originate in the adrenal gland, another 20% in other areas of the abdomen and about 30% in the sympathetic ganglia in the neck, thorax and pelvis (Huddart and Mann, 1991; Buck *et al*, 2001). The most frequent genetic abnormality found in neuroblastoma is the amplification of the MYCN protooncogene, but also other genetic abnormalities have been identified (Maris *et al*, 2007).

Relatively little is known about the aetiology of neuroblastoma. The peak incidence during early childhood indicates that prenatal and perinatal factors may play an important role in its pathogenesis, but the evidence is rather limited and inconclusive (Hamrick *et al*, 2001; Schuz *et al*, 2001). In this study, we aimed to evaluate the impact of birth and parental characteristics on the risk of neuroblastoma, both adrenal and non-adrenal, in a large population-based Norwegian cohort study, using data from the medical birth and cancer registries of Norway.

## Materials and methods

### Study subjects

The Medical Birth Registry of Norway (MBRN) is population-based and contains information on all births in Norway since 1967, defined as all live births and reported foetal deaths of 16 complete weeks of gestation or more. Each record contains information on demographic variables, pregnancy, delivery and the newborn (Irgens, 2000). Data on all deaths registered by Statistics Norway are routinely linked to the birth records. Medical Birth Registry of Norway includes a unique identification number assigned to all live-born children in Norway as well as parents.

Since 1953, the Cancer Registry of Norway (CRN) has received information on all cancer patients in the population. The reporting system is based on pathology and cytology reports, clinical records and death certificates, and provides information about site, histological type and stage of disease at the time of diagnosis (The Cancer Registry of Norway, 2008). Through 1992, registration was based on a modified version of ICD-7. Since 1993, ICD-O has been the basis for coding.

All live-born children in Norway during the period 1967–2004 (*n*=2 127 452) were defined as our study cohort. However, twins, triplets and quadruplets were excluded from the analyses. The personal identification number was used to link the two registries to identify all cases of neuroblastoma in children below 15 years of age. For each child, only the first histologically verified malignant tumour was included in the study. All histologically confirmed neuroblastoma in the SNS were included. Neuroblastomas in the central nervous system and in the eye were excluded. Ganglioneuroblastoma and ganglioneuromas were excluded as well. Each person was followed up from date of birth until 15 years of age, emigration, cancer diagnosis (any site), death or until 31 December 2004. Screening for neuroblastoma has never been introduced in Norway.

### Statistical analysis

Cox proportional hazards regression models (Cox and Oakes, 1984), with time since birth as the time variable, were fitted to obtain relative risk (RR) estimates of neuroblastoma. Tests for trend in the risk of neuroblastoma were performed by including the variables as continuous variables. In subanalyses, the study cohort was stratified by age (below and above 18 months) according to new risk stratification criteria (Maris *et al*, 2007).

The statistical package SPSS (SPSS Inc., 2006) was used for estimating RRs of neuroblastoma with 95% confidence intervals (CIs).

## Results

Altogether 2 127 452 children (1 092 727 boys and 1 034 725 girls) were included in our study, comprising 25 302 860 person-years. The mean time of follow-up was 11.9 years. A total of 178 children (94 boys and 84 girls) were diagnosed with neuroblastoma during 1967–2004. The primary location of 83 tumours was in the adrenal medulla, whereas 95 tumours were located at non-adrenal sites. Ninety-seven cases (54%) were diagnosed before the age of 18 months, 62 cases between 18 months and 4 years, 15 cases between 5 and 9 years and four cases between 10 and 14 years. The number of adrenal and non-adrenal cases was similar before 5 years of age, thereafter there were more cases of non-adrenal origin.

Tables 1 and 2 show results from univariate analyses investigating the relations between birth and parental characteristics and risk of neuroblastoma. There was an increased risk among children with congenital malformations (RR=2.3, 95% CI=1.2–4.8). This increase was restricted to tumours of adrenal origin (RR=3.2, 95% CI=1.3–7.8) and to children diagnosed before the age of 18 months (RR=3.7, 95% CI=1.7–8.0). No other specific association with birth and parental characteristics was observed with regard to location of tumour (adrenal and non-adrenal) (Table 1). Among children having mothers with pre-eclampsia during pregnancy, six developed neuroblastoma during follow-up (RR=1.2, 95% CI=0.5–2.8), all these cases being diagnosed below 18 months of age (RR=2.3, 95% CI=1.0–5.2).

Analyses of the study cohort stratified by age below and above 12 months did not reveal results substantially different from those described above (data not shown).

## Discussion

Analyses of the relations between birth and parental characteristics and the risk of neuroblastoma in a large Norwegian cohort suggested that some of these factors may have an influence. In children below the age of 18 months, there was an increased risk among those with congenital malformations and also a suggestion of increased risk when the mother had pre-eclampsia.

One of the major strengths of this study is the use of large health registries, covering the total population of Norway, to get reliable data on birth and parental characteristics and cancer occurrence. Reporting of cancer cases to CRN has been compulsory since the early 1950s, and the reporting has been almost complete and of high quality (Cancer Registry of Norway, 2007). Medical Birth Registry of Norway is also based on compulsory notification of every birth or late abortion from 16 weeks of gestation onwards. Medical Birth Registry of Norway includes demographic information on the parents, the mother's health before and during pregnancy, complications during pregnancy and delivery, length of pregnancy as well as information on the infant, including birth defects and other perinatal problems (Irgens, 2000).

Within these data sources, we created a large study cohort of 2.1 million children, with a mean follow-up time of almost 12 years, to study the relations between pre- and perinatal factors and neuroblastoma. Nevertheless, only 178 cases were diagnosed, illustrating the rarity of this tumour. Although some estimates different from unity were discovered, the CIs were wide.

In general, children with congenital malformations have an increased risk of cancer (Bjørge *et al*, 2008). In our data, there was an increased risk of neuroblastoma in children with any congenital abnormality. A recent report from the Children's Oncology Group study showed an increased neuroblastoma risk with increasing number of congenital malformations, particularly genitourinary and cardiac anomalies (Menegaux *et al*, 2005). Similar findings were reported from the California Cancer Registry study (Urayama *et al*, 2007). Also other birth defects have been associated with the risk of neuroblastoma (Narod *et al*, 1997). The malformations diagnosed in the eight neuroblastoma cases in our study had the following organ system distribution: heart and blood vessels (one), lip and palate (one), digestive system (two), urinary organs (one), musculoskeletal system (two) and multiple abnormalities (one).

The literature dealing with pre- and perinatal risk factors for neuroblastoma has been largely inconclusive (Hamrick *et al*, 2001; Schuz *et al*, 2001). One recent case-cohort and two relatively recent case-control studies from the US have, however, suggested that certain perinatal factors may be associated with risk. In a study from birth and cancer registries in Minnesota, a maternal history of one fetal loss, maternal drug use and small size for gestational age were associated with neuroblastoma (Johnson *et al*, 2008). In a study from the California Cancer Registry, associations with a number of birth characteristics were observed, including child's race/ethnicity, gestational age/birth weight, caesarean section delivery and maternal pregnancy history (Urayama *et al*, 2007). In another study from the New York State Cancer Registry, pre- and post-term gestations were associated with a significant reduction in risk of neuroblastoma (Buck *et al*, 2001).

In mothers having pre-eclampsia during pregnancy, we found an increased risk of borderline significance in children below the age of 18 months. However, no strong associations have previously been found with diseases/conditions during pregnancy, as pre-eclampsia (Buck *et al*, 2001; Hamrick *et al*, 2001).

In summary, in a huge population-based cohort study, we found that certain birth and parental characteristics may influence the risk of neuroblastoma, but no strong associations were established.

## Figures and Tables

**Table 1:** Relative risk (RR) of neuroblastoma (adrenal and non-adrenal) with 95% confidence intervals (CIs) obtained in Cox regression analyses with age as the time variable, univariate

	**Adrenal medulla**			**Non-adrenal**			**All**			**Total no. of**
	***N***	**RR**	**(95% CI)**	***N***	**RR**	**(95% CI)**	***N***	**RR**	**(95% CI)**	**person-years^a^**
*Sex*										
Male	44	1.0	Referent	50	1.0	Referent	94	1.0	Referent	12 986 975
Female	39	0.9	(0.6–1.4)	45	1.0	(0.6–1.4)	84	0.9	(0.7–1.3)	12 315 885
										
*Season of birth*										
Winter	19	1.0	Referent	22	1.0	Referent	41	1.0	Referent	6 055 108
Spring	21	1.0	(0.5–1.8)	22	0.9	(0.5–1.6)	43	0.9	(0.6–1.4)	6 948 415
Summer	14	0.7	(0.3–1.4)	32	1.4	(0.8–2.4)	46	1.1	(0.7–1.6)	6 377 263
Autumn	29	1.6	(0.9–2.8)	19	0.9	(0.5–1.6)	48	1.2	(0.8–1.8)	5 922 074
										
*Birth year*										
1967–1976	15	1.0	Referent	34	1.0	Referent	49	1.0	Referent	9 048 742
1977–1986	31	2.5	(1.4–4.7)	22	0.8	(0.5–1.4)	53	1.3	(0.9–2.0)	7 367 257
1987–1996	23	1.6	(0.9–3.1)	24	0.8	(0.5–1.3)	47	1.0	(0.7–1.5)	7 103 214
1997–2004	14	1.7	(0.8–3.6)	15	0.9	(0.5–1.6)	29	1.1	(0.7–1.8)	1 783 647
Test for trend			*P*=0.07			*P*=0.4			*P*=0.5	
										
*Gestational age (weeks)*										
<37	3	0.8	(0.3–2.7)	2	0.5	(0.1–2.0)	5	0.6	(0.3–1.6)	1 093 431
37–39	25	1.0	(0.6–1.6)	27	1.0	(0.6–1.5)	52	1.0	(0.7–1.4)	7 533 126
40–41	38	1.0	Referent	43	1.0	Referent	81	1.0	Referent	11 698 199
42+	16	1.5	(0.8–2.7)	14	1.2	(0.6–2.1)	30	1.3	(0.9–2.0)	3 376 235
Test for trend			*P*=0.6			*P*=0.2			*P*=0.2	
										
*Birth weight (g)*										
500–2499	3	1.3	(0.4–4.5)	0	0.0	(0.0-)	3	0.6	(0.2–1.9)	803 494
2500–2999	8	1.1	(0.5–2.5)	11	1.2	(0.6–2.4)	19	1.2	(0.7–2.0)	2 645 261
3000–3499	22	1.0	Referent	28	1.0	Referent	50	1.0	Referent	8 110 752
3500–3999	32	1.3	(0.8–2.2)	36	1.1	(0.7–1.9)	68	1.2	(0.8–1.7)	8 993 123
4000–4499	15	1.4	(0.7–2.7)	18	1.3	(0.7–2.4)	33	1.4	(0.9–2.1)	3 832 887
4500–6300	2	0.8	(0.2–3.4)	1	0.3	(0.0–2.3)	3	0.5	(0.2–1.7)	869 990
Test for trend			*P*=0.6			*P*=0.6			*P*=0.5	
										
*Birth length (cm)*										
40–49	22	0.6	(0.4–1.2)	25	0.8	(0.5–1.5)	47	0.7	(0.5–1.1)	7 514 368
50	23	1.0	Referent	20	1.0	Referent	43	1.0	Referent	5 127 381
51	7	0.3	(0.1–0.8)	21	1.2	(0.6–2.2)	28	0.7	(0.5–1.2)	4 580 532
52	13	0.8	(0.4–1.6)	13	0.9	(0.5–1.8)	26	0.8	(0.5–1.4)	3 634 186
53–60	13	0.8	(0.4–1.5)	13	0.9	(0.4–1.8)	26	0.8	(0.5–1.3)	3 823 916
Test for trend			*P*=0.4			*P*=0.4			*P*=0.2	
										
*Head circumference (cm)* b										
<35	18	1.1	(0.6–1.9)	19	1.2	(0.7–2.1)	37	1.1	(0.7–1.7)	4 508 997
35–36	30	1.0	Referent	29	1.0	Referent	59	1.0	Referent	7 996 344
37+	17	1.5	(0.8–2.7)	12	1.1	(0.6–2.2)	29	1.3	(0.8–2.0)	2 938 088
Test for trend			*P*=0.3			*P*=1.0			*P*=0.5	
										
*Congenital malformations*										
No	78	1.0	Referent	92	1.0	Referent	170	1.0	Referent	24 841 001
Yes	5	3.2	(1.3–7.8)	3	1.6	(0.5–5.1)	8	2.3	(1.2–4.8)	461 860
										
*Maternal age (years)*										
<20	1	0.2	(0.0–1.6)	6	1.2	(0.5–2.9)	7	0.7	(0.3–1.6)	1 326 358
20–24	26	1.0	(0.6–1.7)	25	0.9	(0.5–1.5)	51	1.0	(0.7–1.4)	7 229 682
25–29	34	1.0	Referent	36	1.0	Referent	70	1.0	Referent	8 916 292
30–34	15	0.7	(0.4–1.2)	18	0.8	(0.4–1.4)	33	0.7	(0.5–1.1)	5 394 919
35+	7	0.7	(0.3–1.6)	10	0.9	(0.5–1.9)	17	0.8	(0.5–1.4)	2 419 919
Test for trend			*P*=0.4			*P*=0.6			*P*=0.4	
										
*Parity*										
1	37	1.0	Referent	38	1.0	Referent	75	1.0	Referent	10 431 465
2–3	37	0.8	(0.5–1.3)	45	1.0	(0.6–1.5)	82	0.9	(0.6–1.2)	12 803 473
4–5	8	1.3	(0.6–2.9)	8	1.3	(0.6–2.8)	16	1.3	(0.8–2.3)	1 712 654
6+	1	1.1	(0.2–8.3)	2	2.2	(0.5–9.2)	3	1.7	(0.5–5.4)	255 223
Test for trend			*P*=0.8			*P*=0.9			*P*=0.9	
										
*Pre-eclampsia*										
No	80	1.0	Referent	92	1.0	Referent	172	1.0	Referent	24 639 244
Yes	3	1.3	(0.4–4.2)	3	1.2	(0.4–3.7)	6	1.2	(0.5–2.8)	663 616
										
*Caesarean section*										
No	75	1.0	Referent	87	1.0	Referent	162	1.0	Referent	23 362 347
Yes	8	1.2	(0.6–2.4)	8	1.0	(0.5–2.1)	16	1.1	(0.6–1.8)	1 940 514
										
*Paternal age (years)*										
<25	13	1.1	(0.5–2.1)	22	1.6	(0.9–2.8)	35	1.3	(0.9–2.1)	4 343 748
25–29	33	1.3	(0.8–2.3)	24	0.8	(0.5–1.5)	57	1.1	(0.7–1.6)	8 468 047
30–34	22	1.0	Referent	25	1.0	Referent	47	1.0	Referent	6 959 181
35–39	7	0.6	(0.3–1.5)	18	1.4	(0.8–2.6)	25	1.0	(0.6–1.7)	3 428 839
40+	5	0.8	(0.3–2.2)	4	0.6	(0.2–1.7)	9	0.7	(0.3–1.4)	1 886 960
Test for trend			*P*=0.2			*P*=0.3			*P*=0.09	

a The total number of person-years for some variables may differ due to missing data.

b Head circumference has been recorded since 1978.

**Table 2:** Relative risk (RR) of neuroblastoma with 95% confidence intervals (CIs) obtained in Cox regression analyses, age below and above 18 months, univariate

	**⩽18 months (97 cases)**		**>18 months (81 cases)**	
	**RR**	**(95% CI)**	**RR**	**(95% CI)**
*Sex*				
Male	1.0	Referent	1.0	Referent
Female	0.9	(0.6–1.4)	1.0	(0.6–1.5)
				
*Season of birth*				
Winter	1.0	Referent	1.0	Referent
Spring	1.0	(0.6–1.7)	0.8	(0.4–1.6)
Summer	0.8	(0.4–1.4)	1.5	(0.8–2.9)
Autumn	0.9	(0.5–1.6)	1.6	(0.9–3.0)
				
*Birth year*				
1967–1976	1.0	Referent	1.0	Referent
1977–1986	1.6	(0.9–2.9)	1.1	(0.6–1.9)
1987–1996	1.5	(0.9–2.6)	0.7	(0.4–1.2)
1997–2004	1.3	(0.7–2.4)	1.1	(0.6–2.3)
Test for trend		*P*=0.3		*P*=0.8
				
*Gestational age (weeks)*				
<37	0.6	(0.2–2.0)	0.7	(0.2–2.8)
37–39	0.6	(0.4–1.0)	1.5	(0.9–2.5)
40–41	1.0	Referent	1.0	Referent
42+	1.2	(0.7–2.0)	1.6	(0.8–3.0)
Test for trend		*P*=0.4		*P*=0.5
				
*Birth weight (g)*				
500–2499	1.1	(0.3–3.7)	0.0	(0.0-)
2500–2999	0.9	(0.4–2.1)	1.4	(0.7–2.9)
3000–3499	1.0	Referent	1.0	Referent
3500–3999	1.1	(0.7–1.9)	1.3	(0.8–2.2)
4000–4499	1.8	(1.0–3.1)	0.9	(0.4–1.8)
4500–6300	0.7	(0.2–2.8)	0.4	(0.1–2.8)
Test for trend		*P*=0.3		*P*=1.0
				
*Birth length (cm)*				
40–49	0.7	(0.4–1.3)	0.8	(0.4–1.4)
50	1.0	Referent	1.0	Referent
51	0.7	(0.4–1.4)	0.7	(0.3–1.5)
52	0.6	(0.3–1.3)	1.1	(0.6–2.2)
53–60	0.7	(0.4–1.4)	0.9	(0.5–1.9)
Test for trend		*P*=0.6		*P*=0.2
				
*Head circumference (cm)* a				
<35	1.0	(0.6–1.8)	1.2	(0.7–2.2)
35–36	1.0	Referent	1.0	Referent
37+	1.6	(0.9–2.8)	0.9	(0.4–2.0)
Test for trend		*P*=0.1		*P*=0.5
				
*Congenital malformations*				
No	1.0	Referent	1.0	Referent
Yes	3.7	(1.7–8.0)	0.7	(0.1–4.7)
				
*Maternal age (years)*				
<20	0.6	(0.2–2.0)	0.8	(0.3–2.4)
20–24	1.0	(0.6–1.6)	0.9	(0.6–1.6)
25–29	1.0	Referent	1.0	Referent
30–34	0.9	(0.5–1.5)	0.6	(0.3–1.1)
35+	1.0	(0.5–2.0)	0.6	(0.3–1.5)
Test for trend		*P*=0.9		*P*=0.2
				
*Parity*				
1	1.0	Referent	1.0	Referent
2–3	1.0	(0.6–1.5)	0.8	(0.5–1.3)
4–5	2.0	(1.0–3.8)	0.6	(0.2–1.8)
6+	2.3	(0.6–9.5)	1.1	(0.2–8.0)
Test for trend		*P*=0.2		*P*=0.2
				
*Pre-eclampsia* b				
No	1.0	Referent		
Yes	2.3	(1.0–5.2)		
				
*Caesarean section*				
No	1.0	Referent	1.0	Referent
Yes	1.2	(0.6–2.3)	0.9	(0.4–2.1)
				
*Paternal age (years)*				
<25	1.1	(0.6–1.9)	1.8	(0.9–3.5)
25–29	0.9	(0.5–1.5)	1.4	(0.8–2.6)
30–34	1.0	Referent	1.0	Referent
35–39	0.8	(0.4–1.6)	1.4	(0.7–2.9)
40+	0.6	(0.2–1.5)	0.9	(0.3–2.5)
Test for trend		*P*=0.4		*P*=0.1

a Head circumference has been recorded since 1978.

b All cases were diagnosed below 18 months of age.
